# Enthalpies of mixing of liquid ternary Co–Li–Sn alloys

**DOI:** 10.1007/s00706-014-1284-8

**Published:** 2014-09-24

**Authors:** Andriy Yakymovych, Siegfried Fürtauer, Hans Flandorfer, Herbert Ipser

**Affiliations:** Department of Inorganic Chemistry (Materials Chemistry), University of Vienna, Vienna, Austria

**Keywords:** Thermodynamics, Metals, Calorimetry, Semiempirical calculations

## Abstract

**Abstract:**

The partial and integral molar enthalpies of mixing of liquid Co–Li–Sn alloys were determined using drop calorimetry. The investigations were performed along six sections by the addition of lithium to mixtures with the compositions $x_{\text{Co}}$/$x_{\text{Sn}}$ ≈ 2:98, $x_{\text{Co}}$/$x_{\text{Sn}}$ ≈ 1:9, and $x_{\text{Co}}$/$x_{\text{Sn}}$ ≈ 3:17 as well as by the addition of cobalt to mixtures with the compositions $x_{\text{Li}}$/$x_{\text{Sn}}$ ≈ 3:17, $x_{\text{Li}}$/$x_{\text{Sn}}$ ≈ 1:2, and $x_{\text{Li}}$/$x_{\text{Sn}}$ ≈ 1:1 at a temperature of 1,173 K. The Co–Li–Sn system shows exothermic behavior of the integral molar enthalpy of mixing in the investigated concentration range. The integral molar enthalpy of mixing of liquid Co–Li system was calculated by Miedema’s model to fit our measured ternary data using an extended Redlich–Kister–Muggianu model for substitutional solutions.

**Graphical abstract:**

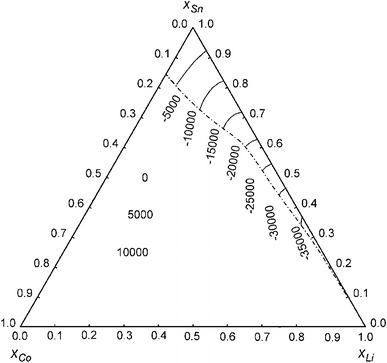

## Introduction

The wide range of industrial applications of lithium-ion batteries (LIBs) initiated extensive research on existing battery elements and also the development of new and alternative materials [1–3]. The three main components of an LIB are the anode, the cathode, and the electrolyte. The process of lithium migration into the anode or cathode is referred to as insertion, and the reverse process, in which lithium moves out of the electrode, is referred to as release. Tin-based intermetallic compounds attracted the continuous attention of investigators due to the larger theoretical electrochemical capacity in the comparison with traditional graphitic materials [4–6]. Among the prospective metallic anode materials are Co–Sn or Co–Sn–C alloys, in which Sn is the main electrochemically active element and Co is responsible for the buffering of volume variations during the Li–Sn alloying–dealloying process [7, 8].

The available literature on the Co–Li–Sn system deals mostly with investigations of the electrochemical reactions of Li with Co–Sn compounds and the corresponding structural changes after initial charge and discharge [9–14]. Most of these authors studied either thin film or nano-crystalline materials. As a basis, however, a reliable and consistent description of the ternary Co–Li–Sn system would be desirable for an understanding of the interaction of Li with anodes based on Co–Sn alloys. To the best of our knowledge, no stable ternary Co–Li–Sn phase has been found in bulk ternary alloys. One of the approaches could be a CALPHAD-type extrapolation based on the known binary systems and supported by experimental thermochemical and phase diagram data for ternary alloys. For this purpose, enthalpies of mixing data for ternary liquid Co–Li–Sn alloys would be highly useful. Therefore, it is the aim of the present paper to investigate experimentally the enthalpies of mixing of liquid Co–Li–Sn alloys. In addition, the interaction parameters for binary Co–Li and ternary Co–Li–Sn systems are evaluated based on Miedema’s model [15] and an extended Redlich–Kister–Muggianu model [16, 17], respectively.

### Literature review: Co–Li

This system is one of the least investigated binary systems. To the best of our knowledge, no experimental thermodynamic data are available for the binary Co–Li system. Moreover, the literature data dedicated to investigations of Co–Li alloys are in considerable disagreement. For example, based on thermal expansion and magnetic measurements Hashimoto [18] suggested a solid solubility of about 30 at. % Li in Co at the transformation temperature, which is in disagreement with Bonnemay et al. [19] who reported a solubility of <0.4 at. % Li. These latter authors reported also the existence of a phase with the stoichiometry Co_3_Li. Furthermore, based on X-ray studies of a Co–Li sample with equiatomic concentration, Magee [20] found lines of an unknown phase that could not be identified. On the other hand, the predicted concentration dependence of the integral molar enthalpy of mixing based on Miedema’s semiempirical model [21] shows endothermic behavior over the entire concentration range instead of exothermic values that would be expected for compound-forming systems [22].

### Co–Sn

Thermodynamic properties of the Co–Sn system are quite well investigated. Several studies are devoted to experimental measurements of the enthalpies of mixing [23–27], and several independent thermodynamic assessments of this binary system [28–31] were performed. In most cases, the authors indicated an S-shaped curve of the integral molar enthalpy of mixing, $\Delta_{\text{mix}}{H}$, versus concentration. However, the reported values of the molar enthalpy of mixing as well as their temperature dependence are highly contradictory. The most recent experimental determination of the mixing enthalpies of liquid Co–Sn alloys between 673 and 1,773 K was performed by Yakymovych et al. [32]. These authors found a significant temperature dependence of the integral molar mixing enthalpy. In contrast to several of the previous experimental studies, the integral molar enthalpy of mixing shows exothermic behavior over the whole concentration range with less negative values at higher temperatures.

### Li–Sn

The enthalpies of mixing of liquid binary Li–Sn alloys were determined by several authors [33–37]. Experimental literature data of the heat of mixing did not reveal any temperature dependence. An extrapolated minimum of $\Delta_{\text{mix}}{H}$ of about −37 kJ mol^−1^ at $x_{\text{Li}}$ = 0.80 could be related with ordering phenomena in the liquid Li–Sn alloys. There are also several assessments of thermodynamic data of the Li–Sn system available, including crystallographic features [38–40]. Based on the available thermodynamic data the phase diagram of Li–Sn system was optimized using the CALPHAD approach [39, 40]. The most recent work regarding the Li–Sn phase diagram combines experimental data (XRD, DTA, $\Delta_{f}{{H^{298} }}$) with a critical evaluation of both thermodynamic and phase diagram information to a self-consistent calculated phase diagram [41].

### Co–Li–Sn

To the best of our knowledge, no experimental thermodynamic data are available for the ternary Co–Li–Sn system.

## Results and discussion

The experimental data of six separate measurements are presented in Tables 1 (sections A, B, and C) and 2 (sections D, E, and F). The tables include information about the number of moles of pure metals dropped into the liquid alloys, drop enthalpy, starting values, and partial and integral molar enthalpies of mixing of investigated alloys.

**Table 1:** Partial and integral molar enthalpies of mixing of liquid Co–Li–Sn alloys along sections A, B, and C at 1,173 K; standard states: pure liquid metals; values on gray background refer to metastable liquid alloys

Dropped mole *n*_*i*_/10^−3^ mol	Measured enthalpy ∆*H*_*Signal*_/J mol^−1^	Partial molar enthalpy		Integral molar enthalpy		
		*x*_i_*	$\Delta_{\text{mix}}{{\bar{H}_{i} }}$/J mol^−1^	$x_{\text{Li}}$	$x_{\text{Sn}}$	$\Delta_{\text{mix}}{{H}}$/J mol^−1^
Section A; $x_{\text{Li}}$ */* $x_{\text{Sn}}$ ≈ 3:17; *i* = Co; *n*_Li_ = 4.0714 × 10^−3 ^mol; *n*_Sn_ = 23.7801 × 10^−3 ^mol; 5 pieces of NIST-sapphire, *k* = 0.5793 ± 0.048 J /µVs^−1^						
0	–	0	–	0.1462	0.8538	−8474 ± 0
0.2814	27,594	0.0050	−21,605 ± 364	0.1447	0.8453	−8,606 ± 4
0.5635	27,538	0.0149	−21,662 ± 364	0.1433	0.8369	−8,735 ± 7
0.8467	27,492	0.0247	−21,707 ± 363	0.1419	0.8286	−8,863 ± 11
1.1402	27,763	0.0344	−21,436 ± 367	0.1404	0.8208	−8,990 ± 14
1.4385	27,554	0.0442	−21,645 ± 364	0.1390	0.8119	−9,119 ± 18
1.7648	27,686	0.0544	−21,513 ± 366	0.1375	0.8029	−9,256 ± 22
2.1053	28,013	0.0649	−21,186 ± 370	0.1359	0.7938	−9,391 ± 26
2.4600	27,861	0.0757	−21,338 ± 368	0.1343	0.7845	−9,531 ± 30
2.8639	27,901	0.0873	−21,298 ± 369	0.1326	0.7742	−9,686 ± 34
3.2893	28,026	0.0994	−21,173 ± 370	0.1307	0.7636	−9,843 ± 39
3.7321	28,378	0.1119	−20,821 ± 375	0.1289	0.7529	−9,997 ± 43
4.2100	28,423	0.1247	−20,776 ± 375	0.1270	0.7417	−10,158 ± 48
4.6998	28,487	0.1378	−20,712 ± 376	0.1251	0.7305	−10,316 ± 53
5.1920	28,342	0.1508	−20,857 ± 374	0.1232	0.7197	−10,473 ± 58
5.7001	28,620	0.1635	−20,579 ± 378	0.1213	0.7088	−10,626 ± 63
6.2142	28,161	0.1762	−21,038 ± 372	0.1195	0.6981	−10,784 ± 68
6.7762	28,375	0.1891	−20,824 ± 375	0.1176	0.6867	−10,946 ± 73
7.4056	28,547	0.2029	−20,652 ± 377	0.1155	0.6745	−11,120 ± 78
8.0921	28,234	0.2176	−20,965 ± 373	0.1133	0.6616	−11,308 ± 84
8.8291	28,272	0.2329	−20,927 ± 373	0.1110	0.6483	−11,501 ± 89
Section B; $x_{\text{Li}}$ */* $x_{\text{Sn}}$ ≈ 1:2; *i* = Co; *n*_Li_ = 17.5263 × 10^−3 ^mol; *n*_Sn_ = 33.9008 × 10^−3 ^mol; 5 pieces of NIST-sapphire, *k* = 0.5815 ± 0.025 J /µVs^−1^						
0	–	0	–	0.3408	0.6592	−18,098 ± 0
0.2722	32,741	0.0026	−16,483 ± 355	0.3390	0.6557	−18,090 ± 2
0.5556	32,473	0.0080	−16,752 ± 352	0.3372	0.6522	−18,082 ± 4
0.8699	32,791	0.0137	−16,434 ± 356	0.3351	0.6482	−18,073 ± 6
1.2257	33,825	0.0200	−15,400 ± 367	0.3329	0.6439	−18,054 ± 8
1.5850	33,561	0.0266	−15,663 ± 364	0.3306	0.6395	−18,038 ± 11
1.9532	34,098	0.0332	−15,126 ± 370	0.3283	0.6351	−18,018 ± 13
2.3440	32,013	0.0401	−17,211 ± 347	0.3259	0.6305	−18,012 ± 16
2.7371	33,088	0.0471	−16,137 ± 359	0.3236	0.6259	−17,999 ± 18
3.1344	29,578	0.0540	−19,646 ± 321	0.3212	0.6213	−18,011 ± 20
3.5337	26,137	0.0609	−23,087 ± 284	0.3189	0.6168	−18,048 ± 22
3.9344	27,639	0.0677	−21,585 ± 300	0.3166	0.6124	−18,073 ± 24
4.3600	27,558	0.0746	−21,666 ± 299	0.3142	0.6077	−18,101 ± 26
4.7860	26,990	0.0816	−22,234 ± 293	0.3118	0.6031	−18,132 ± 28
5.2215	27,672	0.0887	−21,552 ± 300	0.3094	0.5984	−18,158 ± 30
5.6651	26,681	0.0957	−22,543 ± 290	0.3070	0.5938	−18,192 ± 33
6.1172	26,055	0.1028	−23,169 ± 283	0.3046	0.5891	−18,231 ± 34
6.5881	26,406	0.1099	−22,818 ± 287	0.3021	0.5843	−18,269 ± 37
7.0697	26,574	0.1127	−22,650 ± 288	0.2996	0.5795	−18,305 ± 39
7.5561	26,043	0.1245	−23,181 ± 283	0.2971	0.5748	−18,345 ± 41
8.0552	25,539	0.1318	−23,685 ± 277	0.2946	0.5699	−18,390 ± 43
8.5549	25,444	0.1390	−23,781 ± 276	0.2922	0.5652	−18,435 ± 45
9.0585	25,670	0.1462	−23,554 ± 279	0.2898	0.5605	−18,477 ± 46
9.5641	25,649	0.1533	−23,575 ± 278	0.2874	0.5558	−18,520 ± 48
10.1200	25,688	0.1606	−23,537 ± 279	0.2848	0.5508	−18,565 ± 50
10.6792	25,757	0.1682	−23,467 ± 280	0.2822	0.5459	−18,609 ± 53
Section C; $x_{\text{Li}}$ */* $x_{\text{Sn}}$ ≈ 1:1; *i* = Co; *n*_L*i*_ = 10.0778 × 10^−3 ^mol; *n*_Sn_ = 10.0941 × 10^−3 ^mol; 5 pieces of NIST-sapphire, *k* = 0.5783 ± 0.043 J (µVs)^−1^						
0	–	0	–	0.4996	0.5004	−27,759 ± 0
0.1447	38,825	0.0036	−10,331 ± 485	0.4960	0.4968	−27,635 ± 3
0.3043	37,759	0.0110	−11,396 ± 472	0.4922	0.4930	−27,508 ± 7
0.4902	36,236	0.0193	−12,920 ± 453	0.4877	0.4885	−27,377 ± 11
0.7000	33,197	0.0286	−15,959 ± 415	0.4828	0.4836	−27,262 ± 15
0.9116	30,130	0.0384	−19,026 ± 376	0.4780	0.4788	−27,180 ± 19
1.1445	27,149	0.0485	−22,007 ± 339	0.4728	0.4735	−27,123 ± 22
1.3843	27,832	0.0590	−21,324 ± 348	0.4675	0.4683	−27,059 ± 26
1.6268	27,365	0.0694	−21,790 ± 342	0.4623	0.4631	−27,000 ± 29
1.8759	26,990	0.0799	−22,166 ± 337	0.4571	0.4578	−26,945 ± 33
2.1312	26,129	0.0903	−23,027 ± 326	0.4519	0.4526	−26,901 ± 36
2.3945	27,085	0.1008	−22,070 ± 338	0.4466	0.4473	−26,844 ± 40
2.6583	27,254	0.1113	−21,902 ± 340	0.4414	0.4421	−26,787 ± 43
2.9226	27,398	0.1215	−21,758 ± 342	0.4364	0.4371	−26,730 ± 47
3.2011	26,147	0.1318	−23,008 ± 327	0.4312	0.4319	−26,685 ± 50
3.4827	27,041	0.1421	−22,115 ± 338	0.4260	0.4267	−26,631 ± 53
3.7757	27,511	0.1524	−21,645 ± 344	0.4208	0.4215	−26,570 ± 57
4.1038	27,213	0.1634	−21,942 ± 340	0.4151	0.4158	−26,507 ± 61
4.4682	27,088	0.1752	−22,068 ± 338	0.4090	0.4097	−26,442 ± 65
4.8463	27,896	0.1875	−21,260 ± 348	0.4028	0.4035	−26,363 ± 69
5.2242	27,328	0.1997	−21,827 ± 341	0.3968	0.3975	−26,296 ± 73
5.6129	27,351	0.2117	−21,805 ± 342	0.3908	0.3915	−26,228 ± 77
6.0298	27,601	0.2239	−21,555 ± 345	0.3846	0.3852	−26,154 ± 82
6.4696	27,589	0.2365	−21,567 ± 345	0.3783	0.3789	−26,078 ± 86
6.9166	26,767	0.2491	−22,389 ± 334	0.3720	0.3726	−26,017 ± 90
7.3649	27,621	0.2614	−21,535 ± 345	0.3660	0.3666	−25,944 ± 94

* Average of *x*_*i*_ before and after the drop

**Table 2:** Partial and integral molar enthalpies of mixing of liquid Co–Li–Sn alloys along section D, E, and F at 1,173 K; standard states: pure liquid metals; values on gray background refer to metastable liquid alloys

Dropped mole *n*_*i*_/10^−3^ mol	Measured enthalpy ∆*H*_*Signal*_/J mol^−1^	Partial molar enthalpy		Integral molar enthalpy		
		*x*_i_*	$\Delta_{\text{mix}} \bar{H}_{i}$/J mol^−1^	$x_{\text{Co}}$	$x_{\text{Sn}}$	$\Delta_{\text{mix}}{{H}}$/J mol^−1^
Section D; $x_{\text{Co}}$ */* $x_{\text{Sn}}$≈2:98; *i* = Li; *n*_Co_ = 0.9875 × 10^−3^ mol; *n*_Sn_ = 51.4931 × 10^−3^ mol; 5 pieces of NIST-sapphire, *k* = 0.5783 ± 0.043 J (µVs)^−1^						
0	–	0	–	0.0188	0.9812	−331 ± 0
1.6496	−30,113	0.0152	−58,247 ± 376	0.0182	0.9513	−2,096 ± 11
3.0413	−29,707	0.0426	−57,842 ± 371	0.0178	0.9274	−3,493 ± 20
4.6549	−29,310	0.0681	−57,444 ± 366	0.0173	0.9012	−5,017 ± 30
6.2671	−28,287	0.0941	−56,422 ± 353	0.0168	0.8765	−6,427 ± 39
7.8360	−28,938	0.1183	−57,073 ± 362	0.0164	0.8537	−7,745 ± 47
9.7752	−28,502	0.1435	−56,637 ± 356	0.0159	0.8271	−9,268 ± 57
11.6194	−28,150	0.1691	−56,285 ± 352	0.0154	0.8033	−10,620 ± 66
13.4087	−28,400	0.1924	−56,535 ± 355	0.0150	0.7815	−11,867 ± 73
14.9748	−28,072	0.2127	−56,207 ± 351	0.0146	0.7634	−12,897 ± 80
16.8160	−27,118	0.2323	−55,253 ± 339	0.0143	0.7431	−14,022 ± 87
18.6501	−26,777	0.2524	−54,911 ± 335	0.0139	0.7239	−15,076 ± 93
20.5576	−27,333	0.2718	−55,468 ± 341	0.0135	0.7050	−16,131 ± 100
22.5962	−26,495	0.2912	−54,630 ± 331	0.0132	0.6859	−17,177 ± 106
24.8970	−26,168	0.3114	−54,303 ± 327	0.0128	0.6655	−18,281 ± 112
27.2410	−25,875	0.3317	−54,010 ± 323	0.0124	0.6459	−19,331 ± 119
29.6643	−24,644	0.3514	−52,779 ± 308	0.0120	0.6269	−20,318 ± 124
31.3528	−25,440	0.3676	−53,574 ± 318	0.0118	0.6142	−20,988 ± 128
33.4563	−24,561	0.3817	−52,696 ± 307	0.0115	0.5992	−21,764 ± 133
Section E; $x_{\text{Co}}$ */* $x_{\text{Sn}}$ ≈ 1:9; *i* = Li; *n*_Co_ = 17.5263 × 10^−3^ mol; *n*_Sn_ = 33.9008 × 10^−3^ mol; 5 pieces of NIST-sapphire, *k* = 0.6766 ± 0.052 J (µVs)^−1^						
0	–	0	–	0.1015	0.8985	−1,824 ± 0
0.8399	−26,350	0.0109	−52,920 ± 1,438	0.0993	0.8788	−2,940 ± 31
1.7634	−24,845	0.0333	−53,026 ± 1,444	0.0970	0.8582	−4,114 ± 65
2.7546	−25,722	0.0565	−53,903 ± 1,495	0.0946	0.8372	−5,335 ± 100
3.7876	−26,650	0.0798	−54,176 ± 1,511	0.0923	0.8163	−6,553 ± 135
4.8264	−26,989	0.1026	−54,519 ± 1,531	0.0900	0.7963	−7,727 ± 169
6.1173	−25,627	0.1267	−54,332 ± 1,520	0.0873	0.7728	−9,102 ± 209
7.4514	−27,528	0.1525	−55,202 ± 1,571	0.0848	0.7500	−10,466 ± 249
8.8633	−25,863	0.1779	−55,002 ± 1,559	0.0822	0.7272	−11,818 ± 289
10.3342	−26,806	0.2030	−54,986 ± 1,558	0.0797	0.7049	−13,142 ± 328
11.981	−27,310	0.2285	−55,490 ± 1,587	0.0770	0.6815	−14,548 ± 370
13.6349	−27,617	0.2537	−55,797 ± 1,605	0.0745	0.6595	−15,878 ± 409
15.3609	−26,096	0.2779	−55,845 ± 1,608	0.0721	0.6380	−17,180 ± 449
17.0941	−28,435	0.3011	−56226 ± 1,630	0.0698	0.6178	−18,416 ± 486
18.8431	−26,926	0.3230	−55,107 ± 1,565	0.0677	0.5987	−19,553 ± 519
20.706	−26,602	0.3443	−54,783 ± 1,546	0.0655	0.5796	−20,677 ± 552
22.5731	−26,038	0.3649	−54,219 ± 1,513	0.0635	0.5616	−21,718 ± 582
24.4446	−27,129	0.3843	−54,225 ± 1,514	0.0616	0.5447	−22,698 ± 610
26.3204	−26,002	0.4027	−54,182 ± 1,511	0.0598	0.5287	−23,621 ± 636
28.2337	−26,727	0.4201	−54,024 ± 1,502	0.0580	0.5134	−24,504 ± 662
30.1859	−25,363	0.4369	−53,543 ± 1,474	0.0563	0.4986	−25,340 ± 685
32.3296	−26,620	0.4536	−53,223 ± 1,456	0.0546	0.4833	−26,194 ± 709
34.4749	−24,848	0.4701	−53,029 ± 1,444	0.0530	0.4689	−26,993 ± 731
36.6503	−25,344	0.4857	−53,214 ± 1,455	0.0514	0.4552	−27,761 ± 752
39.0203	−24,559	0.5012	−52,740 ± 1,427	0.0499	0.4411	−28,533 ± 773
Section F; $x_{\text{Li}}$ */* $x_{\text{Sn}}$ ≈ 3:17; *i* = Li; *n*_Co_ = 6.3543 × 10^−3^ mol; *n*_Sn_ = 35.4535 × 10^−3^ mol; 5 pieces of NIST-sapphire, *k* = 0.5739 ± 0.024 J (µVs)^−1^						
0	–	0	–	0.1522	0.8478	−2,746 ± 0
0.5648	−3,862	0.0067	−32,043 ± 52	0.1502	0.8364	−3,137 ± 1
1.1641	−5,458	0.0202	−33,638 ± 74	0.1481	0.8248	−3,563 ± 2
1.8398	−10,545	0.0347	−38,725 ± 143	0.1458	0.8120	−4,108 ± 4
2.5414	−12,609	0.0498	−40,790 ± 171	0.1435	0.7991	−4,689 ± 7
3.2618	−14,135	0.0649	−42,316 ± 191	0.1412	0.7863	−5,291 ± 10
4.0513	−14,658	0.0805	−42,839 ± 199	0.1388	0.7728	−5,939 ± 13
4.8494	−17,031	0.0963	−45,212 ± 231	0.1364	0.7595	−6,611 ± 17
5.6591	−17,571	0.1117	−45,752 ± 238	0.1341	0.7465	−7,280 ± 20
6.4890	−16,481	0.1270	−44,662 ± 223	0.1318	0.7337	−7,923 ± 24
7.3289	−17,285	0.1419	−45,466 ± 234	0.1295	0.7211	−8,566 ± 27
8.1847	−18,918	0.1566	−47,099 ± 256	0.1273	0.7088	−9,226 ± 31
9.0794	−17,234	0.1713	−45,415 ± 233	0.1250	0.6963	−9,863 ± 35
9.9827	−18,106	0.1858	−46,287 ± 245	0.1229	0.6841	−10,499 ± 39
10.8976	−18,576	0.2000	−46,757 ± 252	0.1207	0.6723	−11,129 ± 42
11.9162	−18,589	0.2145	−46,769 ± 252	0.1184	0.6595	−11,806 ± 46
12.9535	−18,735	0.2294	−46,916 ± 254	0.1162	0.6470	−12,472 ± 50
13.9994	−19,524	0.2440	−47,705 ± 264	0.1140	0.6348	−13,133 ± 54
15.0555	−19,653	0.2581	−47,834 ± 266	0.1119	0.6230	−13,778 ± 58
16.2124	−19,143	0.2724	−47,323 ± 259	0.1097	0.6106	−14,448 ± 62
17.388	−18,138	0.2869	−46,319 ± 246	0.1075	0.5985	−15,081 ± 66
18.5679	−18,211	0.3010	−46,391 ± 247	0.1054	0.5868	−15,694 ± 69
19.7522	−18,209	0.3145	−46,390 ± 247	0.1033	0.5755	−16,285 ± 73
21.0171	−17,036	0.3280	−45,216 ± 231	0.1013	0.5639	−16,868 ± 76
22.4247	−17,232	0.3422	−45,413 ± 233	0.0990	0.5515	−17,494 ± 79
23.9519	−17,697	0.3570	−45,878 ± 240	0.0967	0.5387	−18,154 ± 83

* Average of *x*_*i*_ before and after the drop

The Li–Sn alloys are in the liquid state over the whole concentration range at 1,173 K [42]; therefore, the starting compositions on the Li–Sn side were chosen over a large concentration interval: Li_0.15_Sn_0.85_, Li_0.33_Sn_0.67_, Li_0.50_Sn_0.50_, and Li_0.75_Sn_0.25_. However, measurements along the section $x_{\text{Li}}$/$x_{\text{Sn}}$ ≈ 3:1 were unsuccessful. In the case of the Co–Sn system, only alloys with Co contents up to about 17 at. % are liquid at the investigated temperature [43]; taking this into account, Co_0.02_Sn_0.98_, Co_0.10_Sn_0.90_, and Co_0.15_Sn_0.85_ were taken as the starting alloys for the present investigations. The starting values of $\Delta_{\text{mix}}{H}$ for the binary Li–Sn and Co–Sn subsystems required for the evaluation of the integral molar enthalpy of mixing for ternary liquid Co–Li–Sn alloys were directly taken from recent investigations [32, 36].

**Fig. 1:**
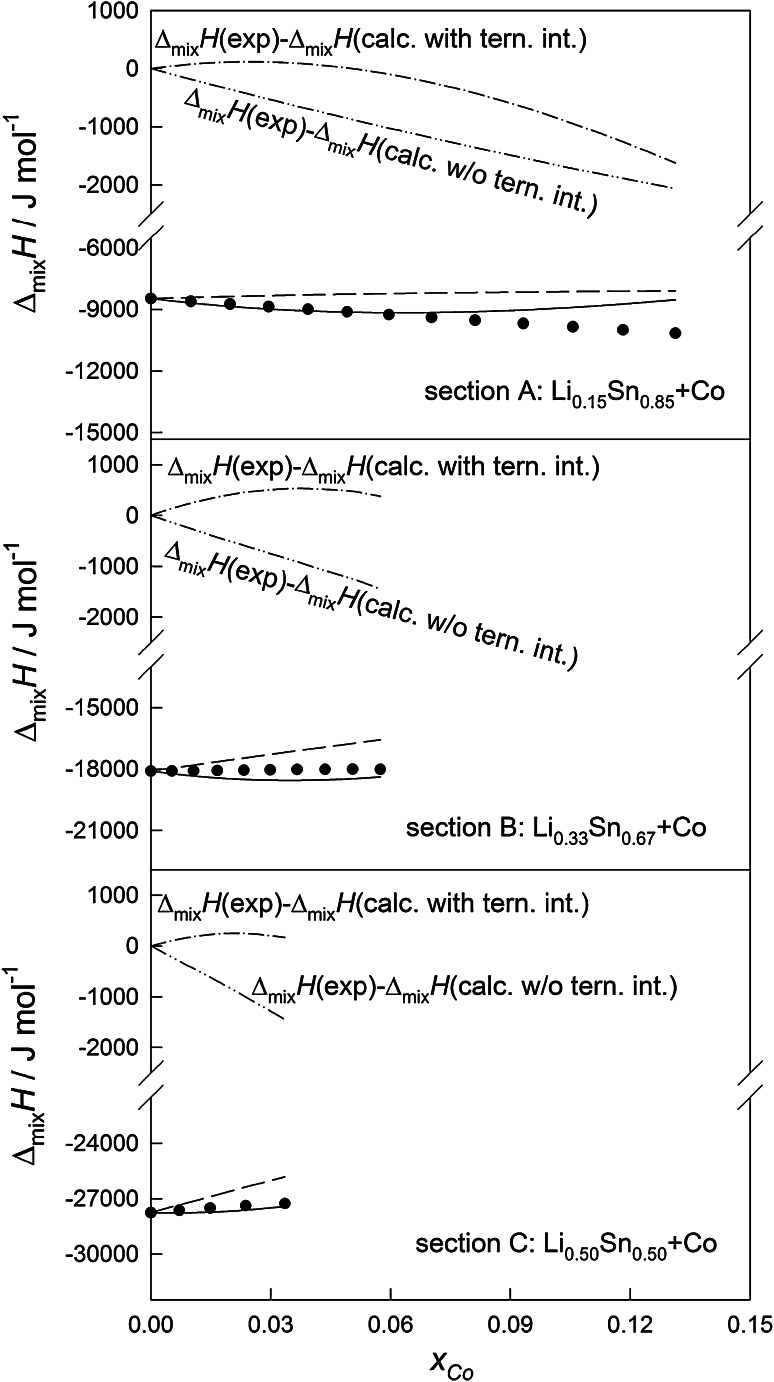
Integral and partial molar enthalpy of mixing of liquid Co–Li–Sn alloys at 1,173 K for the sections A ($x_{\text{Li}}$ */* $x_{\text{Sn}}$ ≈ 3:17), B ($x_{\text{Li}}$ */* $x_{\text{Sn}}$ ≈ 1:2), and C ($x_{\text{Li}}$ */* $x_{\text{Sn}}$ ≈ 1:1) (*filled circle* experiment, *short* *dashed* calculated without ternary interactions; *continuous dashed* calculated with ternary interactions)

**Fig. 2:**
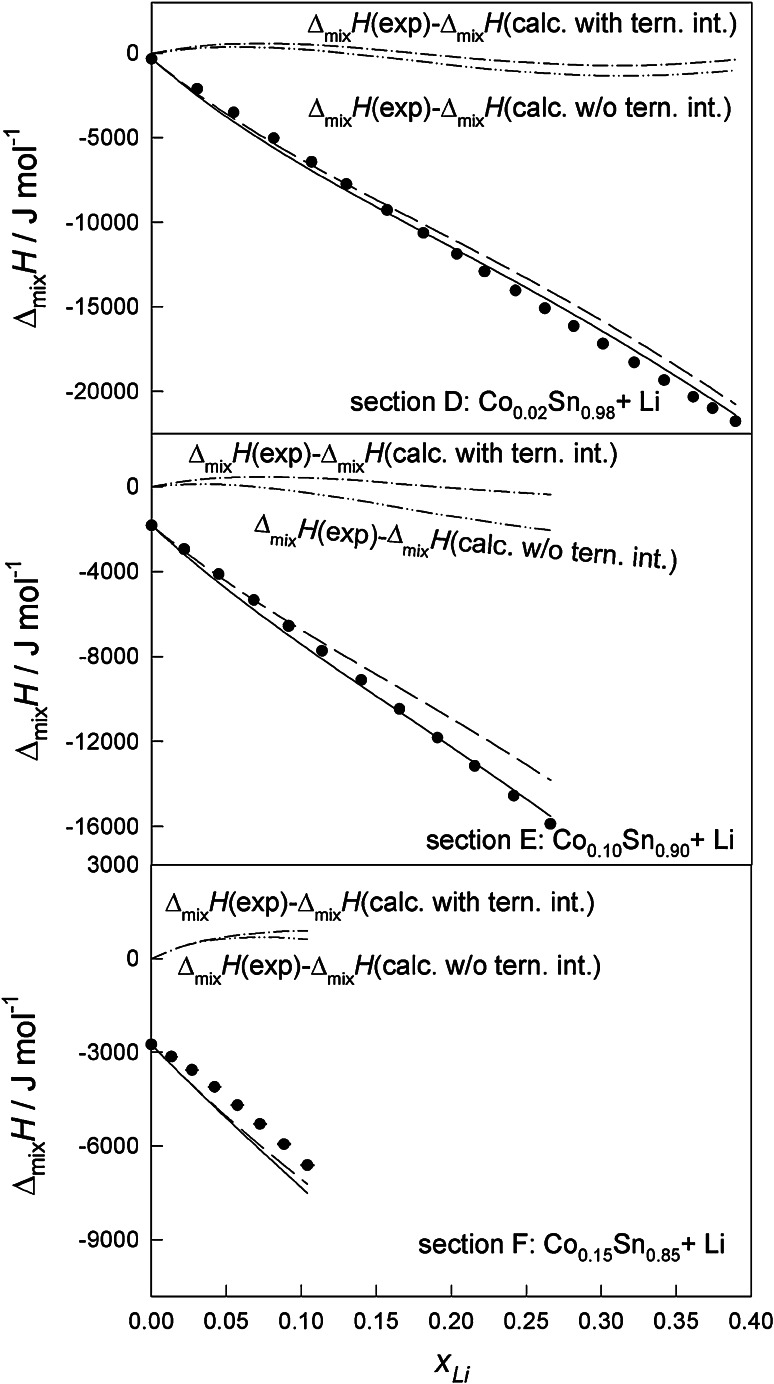
The integral molar enthalpy of mixing of Co–Li–Sn alloys at 1,173 K for the sections D ($x_{\text{Co}}$ */* $x_{\text{Sn}}$ ≈ 2:98), E ($x_{\text{Co}}$ */* $x_{\text{Sn}}$ ≈ 1:9), and F ($x_{\text{Co}}$ */* $x_{\text{Sn}}$ ≈ 3:17) (*filled circle* experiment, *short dashed* calculated without ternary interactions, *continuous dashed* calculated with ternary interactions)

Experimental integral molar enthalpies of mixing were plotted versus concentration of Li or Co and are shown in Figs. 1 and 2, respectively. The obtained results indicate that enthalpies of mixing in the investigated concentration range show exothermic behavior. The negative values of the integral molar enthalpy of mixing indicate preferred interactions between unlike kinds of atoms in the liquid state.

**Fig. 3:**
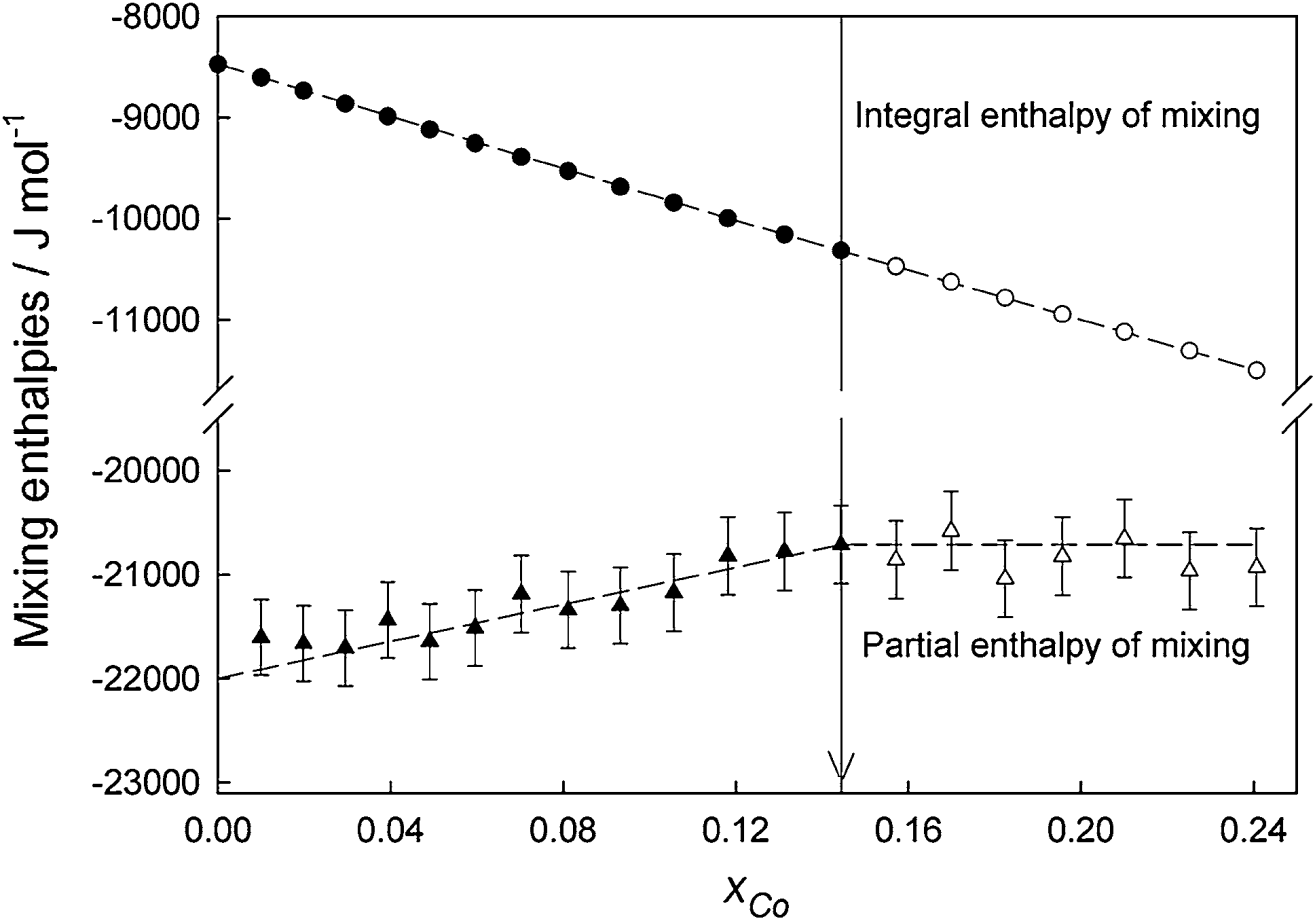
The concentration dependence of the partial and integral molar enthalpy of mixing along section A ($x_{\text{Li}}$ */* $x_{\text{Sn}}$ ≈ 3:17)

Crossing the liquidus line entering into a multiphase field is usually indicated by a kink in the composition dependence of the integral molar enthalpy of mixing and by a corresponding change of the partial molar enthalpy values. Depending on the material dropped and the additional phases formed this change is more or less accompanied by a discontinuity. As an example, the course of the integral and partial molar enthalpies of mixing along section A (pure Co dropped into liquid Li_0.15_Sn_0.85_ alloy) is shown in Fig. 3.

In this case no discontinuity but a clear kink followed by rather constant values appears in the course of the partial molar enthalpies of mixing. The constant partial values indicate a transition into a multiphase region, which occurred after a number of drops of Co into the liquid Li_0.15_Sn_0.85_ alloy. The corresponding points indicating the liquidus boundary were determined only based on the partial molar enthalpy of mixing data and added to Fig. 5. The values within the shadowed fields in Tables 1 and 2 are valid for compositions outside the homogeneous liquid phase.

For a mathematical description of the composition dependence of the integral molar enthalpy of mixing of liquid Co–Li–Sn alloys, the experimental data were subjected to a least-squares fit based on a Redlich–Kister–Muggianu polynomial [16, 17]:

1 $$\Delta_{\text{mix}}{H} = \sum\limits_{i} {\sum\limits_{j > i} {\left[ {x_{i} x_{j} \sum\limits_{v} {^{v} L_{i,j}^{H} \left( {x_{i} - x_{j} } \right)^{v} } } \right]} } + x_{i} x_{j} x_{k} \left( {^{(0)} L_{i,j,k}^{H} x_{i} +^{(1)} L_{i,j,k}^{H} x_{j} +^{(2)} L_{i,j,k}^{H} x_{k} } \right)$$

where *i*, *j*, *k* are equal to 1, 2, 3 for the elements Co, Li, and Sn, respectively; ${}^{v}L_{i,j}^{H} \left( {v = \, 0,{ 1},{ 2}, \ldots } \right)$ are the interaction parameters of the three binary systems; ${}^{\alpha }L_{i,j,k}^{H} \left( {\alpha = \, 0,{ 1},{\text{ and 2}}} \right)$ are three ternary interaction parameters; *x*_*i*_, *x*_*j*_, *x*_*k*_ are the mole fractions of ternary alloys. For this evaluation, it is necessary to know the interaction parameters ${}^{v}L_{i,j}^{H}$ of the binary subsystems which are available in literature for the systems Li–Sn and Co–Sn [32, 36].

Since no literature values are available for the Co–Li system, the corresponding integral molar enthalpy of mixing was estimated using Miedema’s model [44] similar to Refs. [45, 46]:

2 $$\Delta_{\text{mix}}{H} = x_{i} x_{j} \left( {x_{i}^{s} \Delta H_{j,i}^{0} + x_{j}^{s} \Delta H_{i,j}^{0} } \right)$$

where the enthalpy of solution of liquid *i* in liquid *j* at infinite dilution for a binary alloy, $\Delta H_{i,j}^{0}$, is expressed as:

3 $$\Delta H_{i,j}^{0} = \frac{{2PV_{i}^{2/3} }}{{n_{i}^{ - 1/3} + n_{j}^{ - 1/3} }}\left[ { - \left( {\Delta \varPhi^{*} } \right)^{2} + \frac{Q}{P}\left( { \Delta n^{1/3} } \right)^{2} - \frac{R}{P}} \right]$$

where *V*_*i*_, Φ*, and *n* ^1/3^ are the parameters; *P*, *Q*, and *R* are the constants determined by Miedema [44]. The *R/P* value for liquid alloys, *R* ^*^ */P*, was calculated by multiplying the quotient for solid alloys, *R/P*, by a factor of 0.73.

According to Miedema’s model, the $x_{i}^{s}$ term in Eq. (5) is given as.

4 $$x_{i}^{s} = \frac{{x_{i} V_{i}^{2/3} }}{{x_{i} V_{i}^{2/3} + x_{j} V_{j}^{2/3} }}$$

The parameter values used for the evaluation of the Co–Li system were taken from Niessen et al. [22], and the molar volume values *V*_*i*_ were taken from Iida and Guthrie [47]. All binary and ternary interaction parameters are listed in Table 3.

**Table 3:** Binary and ternary interaction parameters in liquid Co–Li–Sn system at 1,173 K

System	Literature	Interaction parameters/J mol^−1^
Co–Li	Present work	${}^{0}L{}_{\text{Co, Li}}^{H}$ = 31,822.096
		${}^{1}L{}_{\text{Co, Li}}^{H}$= 971.211
Co–Sn	[32]	${}^{0}L{}_{\text{Co, Sn}}^{H}$ = −30,032.9453
		${}^{1}L{}_{\text{Co, Sn}}^{H}$ = −12,595.8043
Li–Sn	[36]	${}^{0}L{}_{\text{Li, Sn}}^{H}$ = −111,137
		${}^{0}L_{\text{Li, Sn}}^{H}$ = −89,726
Co–Li–Sn	Present work	${}^{0}L_{Co,Li,Sn}^{H}$ = 1,240,537
		${}^{1}L_{Co,Li,Sn}^{H}$ = −295,725
		${}^{2}L_{Co,Li,Sn}^{H}$ = −216,998

Based on Eq. (1) the integral molar enthalpies of mixing were calculated for the investigated ternary composition range. Calculated integral molar enthalpy curves for all sections have been plotted and are shown in Figs. 1 and 2. According to comparative analysis, the deviations between $\Delta_{\text{mix}}{H}$ data fitted using the Redlich–Kister–Muggianu polynomial and experimental values for all investigation sections are <1 kJmol^−1^. By the comparison of this number with absolute values of the obtained molar enthalpy of mixing we found satisfactory agreement of the presented results. The deviation between $\Delta_{\text{mix}}{H}$ values calculated by Eq. (1) without the terms for ternary interactions and experimental data reaches approx. 2 kJ mol^−1^. Nevertheless, the presented disagreement between experimental and calculated data is not significant enough to assume real ternary interactions in the liquid.

**Fig. 4:**
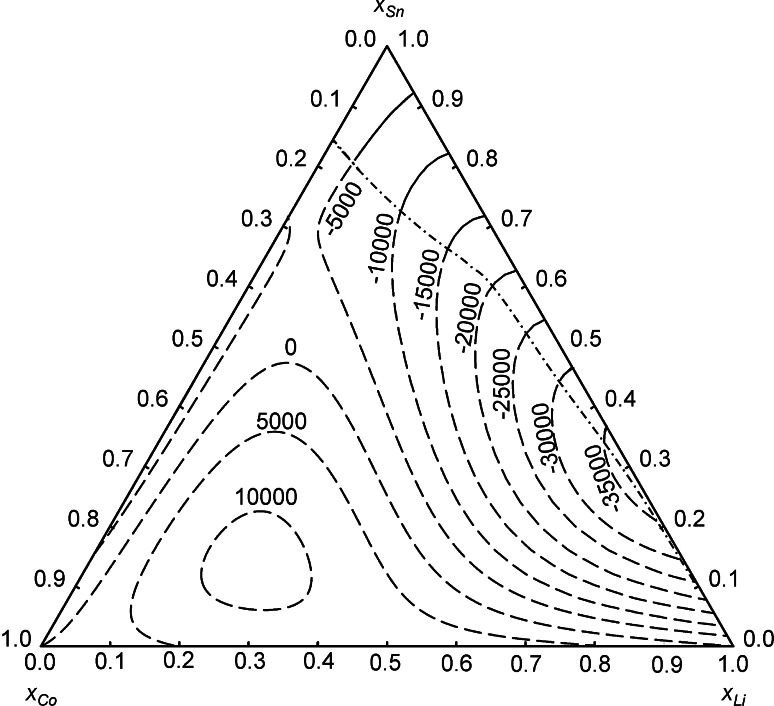
Isoenthalpy curves of liquid Co–Li–Sn alloys at 1,173 K; standard states: pure liquid metals. *Dashed lines* correspond to metastable liquid alloys

Figure 4 shows isoenthalpy curves across the entire ternary composition range plotted in a Gibbs triangle. The values outside of the homogeneous liquid range at 1,173 K, which are shown as dashed curves, have to be considered as integral molar enthalpies of the metastable liquid phase. According to this plot, the ternary system shows an exothermic enthalpy minimum of approximately −35 kJ mol^−1^ in the Li–Sn binary system, and a maximum of approximately +10 kJ mol^−1^ in the Co-rich corner of the ternary system. Considering the rather small fully liquid region and the large area of extrapolated values, the latter ones are of limited significance.

**Fig. 5:**
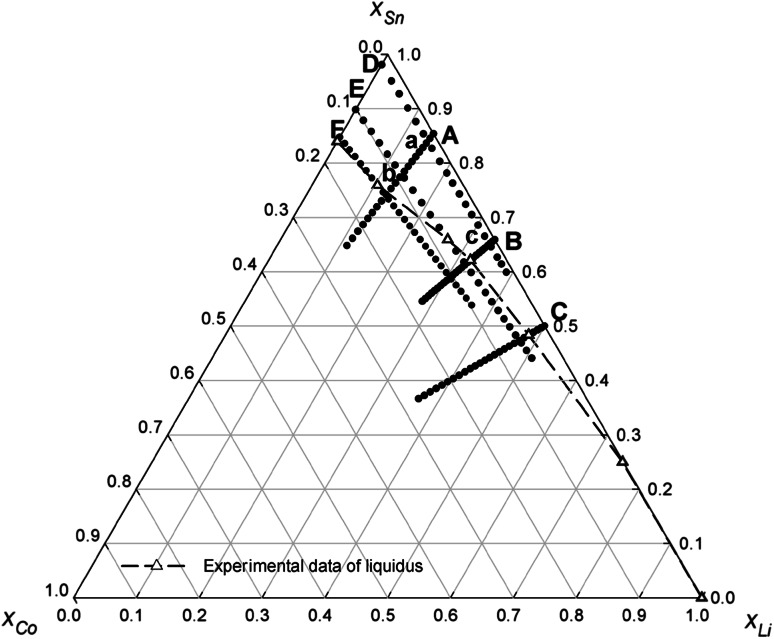
Measured sections and alloy compositions in the ternary Co–Li–Sn system at 1,173 K: (*A*) $x_{\text{Li}}$/$x_{\text{Sn}}$ ≈ 3:17, (*B*) $x_{\text{Li}}$/$x_{\text{Sn}}$ ≈ 1:2, (*C*) $x_{\text{Li}}$/$x_{\text{Sn}}$ ≈ 1:1, (*D*) $x_{\text{Co}}$/$x_{\text{Sn}}$ ≈ 2:98, (E) $x_{\text{Co}}$/$x_{\text{Sn}}$ ≈ 1:9, and (*F*) $x_{\text{Co}}$/$x_{\text{Sn}}$ ≈ 3:17; the estimated liquidus limit is marked by the *dashed line*

Further proof of the quality of our data is the good agreement of values from different experiments close to the three intersection points a, b, and c of the four concentration sections A and B with D and E (see Table 4; Fig. 5). The maximum errors are <1 kJ mol^−1^ which is satisfying taking into account the method applied and the delicate alloy system.

**Table 4:** Experimental values of the integral molar enthalpy of mixing at the intersection points **a**, **b**, and **c**

Intersection	Composition			Integral enthalpy of mixing^a^/J mol^−1^				Eq. (1)
	$x_{\text{Co}}$	$x_{\text{Li}}$	$x_{\text{Sn}}$	A (*x*_Li_/*x*_Sn_ ≈ 3:17)	B (*x*_Li_/*x*_Sn_ ≈ 1:2)	C (*x*_Co_/*x*_Sn_ ≈ 2:98)	E (*x*_Co_/*x*_Sn_ ≈ 1:9)	
**a**	0.0161	0.1438	0.8401	−8,700 ± 50		−8,450 ± 100		−8,800
**b**	0.0867	0.1335	0.7798	−9,600 ± 50			−8,800 ± 200	−9,050
**c**	0.0125	0.3361	0.6514		−18,100 ± 50	−19,050 ± 150		−18,400

^a^Rounded to 50 J mol^−1^

## Experimental

The samples were prepared from cobalt foil (99.9+%, Alfa Aesar, Karlsruhe, Germany), tin ingot (99.998 %, Alfa Aesar, Karlsruhe, Germany), and lithium wire (99.8 %, Alfa Aesar, Karlsruhe, Germany). The lithium wire was cleaned in a supersonic bath in *n*-hexane and the solvent removed under vacuum in the glove box antechamber. The copper foil was treated under H_2_ flow at 473 K for 5 h to remove any oxide layers. The tin rods were cleaned with a piece of fine sandpaper before using. All operations with Li were performed in a glove box (M. Braun, LabMaster 130) with an atmosphere of purified Ar inside (O_2_ and H_2_O <5 ppm each). Pieces of Li that were used for dropping into the calorimeter were placed into the drop chamber within the glove box which was then transferred to the calorimeter using an argon-filled plastic bag.

The measurements were performed with a high-temperature Calvet-type microcalorimeter HT-1000 (Setaram, Lyon, France). A detailed description of the experimental setup of this calorimeter was given by Flandorfer et al. [48]. All experiments were performed under a continuous gas flow of pure Ar (approx. 30 cm^3^/min; 5 N, further purified from oxygen). Mo-crucibles (inner diameter 9 mm, length 80 mm) served as sample containers because Mo is inert against liquid Li at the investigated temperature range. The interval between individual drops was 40 min. The obtained signals were recorded with an acquisition interval of 0.5 s. Drops of NIST standard α-Al_2_O_3_ (National Institute of Standards and Technology, Gaithersburg, MD) were used for the determination of the calorimeter constant (calibration of the heat flow) at the end of each series of measurements. For the control of the experiments and the evaluations of the obtained data the programs LabView and HiQ were used. The measured enthalpy ∆*H*_Signal_ (integrated heat flow at constant pressure) is given by.

5 $$\Delta H_{\text{Signal}} = n_{i} (H_{{m,i,T_{\text{M}} }} - H_{{m,i,T_{\text{D}} }} ) + \Delta H_{\text{Reaction}} \,,$$

where *n*_*i*_ is the number of moles of the added sample, *H*_*m*_ denotes molar enthalpies, *T*_D_ is the drop temperature (usually 298 K), and *T*_M_ is the calorimeter temperature of the respective measurement in K. The molar enthalpy difference $(H_{{m,\;i,\;T_{\text{M}} }} - H_{{m,\;i,\;T_{\text{D}} }} )$ was calculated using the SGTE database for pure elements [49]. Because of the rather small masses of added samples, the partial molar enthalpy of mixing values can be approximately calculated as.

6 $$\Delta_{\text{mix}}{{\bar{H}_{i} }} = \frac{{\Delta H_{\text{Reaction}} }}{{n_{i} }}\,.$$

The integral molar enthalpy of mixing is calculated by.

7 $$\Delta_{\text{mix}}{H} = \frac{{\sum {\Delta H_{\text{Reaction}} } }}{{\left( {n_{j} + \sum\nolimits_{i} {n_{i} } } \right)}},$$

where *n*_*j*_ is the molar amount of the metal sample in the crucible before dropping.

The measurement temperature for the Co–Li–Sn system was 1,173 K, corresponding to the limit of safe handling of liquid Li. Furthermore, at higher temperatures Li-rich melts started creeping out of the crucible and reacting with the wall of the outer quartz glass tube. For the experiments, pieces of pure Li were dropped into Co–Sn mixtures with $x_{\text{Co}}$/$x_{\text{Sn}}$ ≈ 2:98, $x_{\text{Co}}$/$x_{\text{Sn}}$ ≈ 1:9, and $x_{\text{Co}}$/$x_{\text{Sn}}$ ≈ 3:17, and pieces of Co were dropped into Li–Sn mixtures with $x_{\text{Li}}$/$x_{\text{Sn}}$ ≈ 3:17, $x_{\text{Li}}$/$x_{\text{Sn}}$ ≈ 1:2, and $x_{\text{Li}}$/$x_{\text{Sn}}$ ≈ 1:1, according to the compositions shown in Fig. 5.

In addition, calorimetric measurements were also performed by the addition of pure Co to a mixture $x_{\text{Li}}$/$x_{\text{Sn}}$ ≈ 3:1 (two separate runs) and by addition of Co to pure liquid Li (four separate runs) at 1,173 K. From the results of these measurements, it had to be concluded that Co does not react with liquid Li or Li-rich Li–Sn alloys at 1,173 K. Thus, any further experiments in this composition range were abandoned.

Considering the numerous calibration measurements done by dropping NIST standard sapphire, the standard deviation can be estimated to be <±1 %. The overall error of the measured enthalpy is about ±150 J.

Unfortunately, it was not possible to check the phases formed after the calorimetric measurements due to the rather rapid oxidation of the samples. Contrary to the filling of the drop chamber (see above), it takes some time to remove the crucible with the alloy from the calorimeter and transfer it to an oxygen-free environment. Therefore, X-ray diffraction measurements showed the presence of binary and ternary oxides in the alloys. Nevertheless, since any handling before and during the calorimetric measurements was done under purified argon it can be assumed that the obtained enthalpy values should be reliable.
